# Supplementary material for: Splice-Junction-Based Mapping of Alternative Isoforms in the Human Proteome
Source: Cell Rep. Author manuscript; Available in PMC 2020 Jan 15. (PMC6961840; doi:10.1016/j.celrep.2019.11.026)
Supplement: 2 [file NIHMS1546469-supplement-2.zip › DF1/PXD010154/Liver/Liver_43_NDUFS7_GSHIPVPDLMWVQASGSGR.pdf]

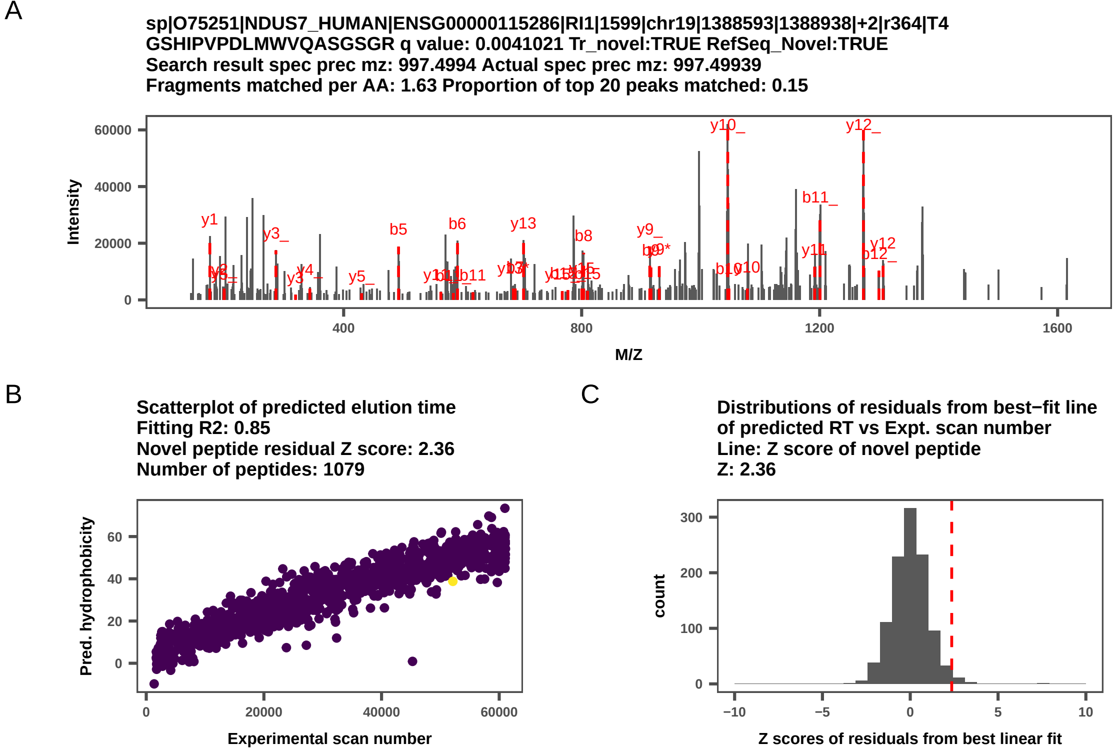
A
sp|O75251|NDUS7_HUMAN|ENSG00000115286|RI1|1599|chr19|1388593|1388938|+2|r364|T4
GSHIPVPDLMWVQASGSGR q value: 0.0041021 Tr_novel:TRUE RefSeq_Novel:TRUE
Search result spec prec mz: 997.4994 Actual spec prec mz: 997.49939
Fragments matched per AA: 1.63 Proportion of top 20 peaks matched: 0.15
Intensity
60000
40000
20000
0
400
800
1200
1600
M/Z
y1
y3_
y4_
y5_
y3
b5
b6
b11
y13
b8
y9_
y10_
b10
y10
b11_
y11
y12_
y12
b12_
B
Scatterplot of predicted elution time
Fitting R2: 0.85
Novel peptide residual Z score: 2.36
Number of peptides: 1079
Pred. hydrophobicity
60
40
20
0
0
20000
40000
60000
Experimental scan number
C
Distributions of residuals from best−fit line
of predicted RT vs Expt. scan number
Line: Z score of novel peptide
Z: 2.36
count
300
200
100
0
−10
−5
0
5
10
Z scores of residuals from best linear fit
